# Comparison of multicolor scanning laser ophthalmoscopy and optical coherence tomography angiography for detection of microaneurysms in diabetic retinopathy

**DOI:** 10.1038/s41598-021-96371-y

**Published:** 2021-08-23

**Authors:** Takato Sakono, Hiroto Terasaki, Shozo Sonoda, Ryoh Funatsu, Hideki Shiihara, Eisuke Uchino, Toshifumi Yamashita, Taiji Sakamoto

**Affiliations:** 1 Department of Ophthalmology, Kagoshima University Graduate School of Medical and Dental Sciences, Kagoshima, Japan; 2 Kagoshima Sonoda Eye and Plastic Surgery Clinic, Kagoshima, Japan

**Keywords:** Eye diseases, Retinal diseases

## Abstract

This study aimed to evaluate the usefulness of multicolor (MC) scanning laser ophthalmoscopy (MC-SLO) in detecting microaneurysm (MA) in eyes with diabetic retinopathy (DR). This was a retrospective cross-sectional study. Eyes with DR underwent fluorescein angiography (FA), MC-SLO, optical coherence tomography angiography (OCTA), and color fundus photography (CFP) were analyzed. The foveal region was cut in an 6 × 6 mm image and the number of MA in each image was counted by retina specialists to determine the sensitivity and positive predictive value. FA results were used as the ground standard. MAs were classified as those with early, late, or no dye leakage based on FA images. Fifty-four eyes of 35 patients with an average age of 64.5 ± 1.24 years were included. The sensitivity of MA detection was 37.3%, 15.3%, and 4.12% in MC-SLO, OCTA, and CFP, respectively (P < 0.01 in each pair).The positive predictive value was 66.4%, 46.4%, and 27.6% in MC, OCTA, and CFP, respectively (P < 0.01 in each pair). Sensitivity for MAs with early leakage was 36.4% in MC-SLO, which was significantly higher than 4.02% in OCTA. MC-SLO was more useful in detecting MA in eyes with DR than OCTA.

## Introduction

Diabetic macular edema (DME) is a major cause of poor vision in patients with diabetic retinopathy^1^. The major cause of DME is vascular hyperpermeability, which is associated with damage of vascular endothelial cells, formation of microaneurysms (MA), and secretion of vascular endothelial growth factor (VEGF)^2–7^. Recently, intravitreous injection of anti-VEGF drugs is the main treatment for DME^6,7^. However, direct photocoagulation of MAs is sometimes effective in DME^8–14^. Because photocoagulation of MA can reduce the number of the anti-VEGF therapies^10,11^, it is beneficial for patients, the medical staff, and the medical economy.

Currently, fluorescein angiography (FA) is the gold standard of MA detection^7,10,11^. Although MAs could be clearly detected using FA, it has the major drawback of invasiveness^15,16^. Therefore, it is difficult to repeatedly perform in the evaluation of the treatment effect.

Recently, it has been known that MAs can be detected by optical coherence tomography angiography (OCTA) noninvasively. However, its detection rate is lower than that of FA (41–64% compared with that of FA)^17–20^. Moreover, in OCTA, MA is not always shown as a dot-like hyperfluorescence as in FA, but there are variations in the appearance, such as capillary loops, dilated capillary segments, and focal dilatation^10^. Thus, the specificity of MAs is 75% compared with that of FA, which is low probably because it is difficult to distinguish from normal capillaries^20^.

A multicolor (MC) scanning laser ophthalmoscope (MC-SLO) is a new device that creates images using three different wavelength lasers, such as blue, green, and red^21^. The blue wavelength laser, red wavelength laser, and green wavelength laser reaches the superficial, deep, and intermediate layers of the retina, respectively^22^. Because images are created for each layer, the image merged by these three images could reveal the retinal structure more clearly than a normal color fundus photograph (CFP). On the basis of these characteristics, it has already been reported that MC-SLO is superior to CFP in detecting retinal diseases, including epiretinal membrane and geographic atrophy^23–27^.

In our preliminary observation, we found that MAs showed a characteristic morphology in MC-SLO. Additionally, because normal retinal capillaries do not appear in MC as in OCTA, MA can be identified more easily and has high specificity in MC-SLO. Therefore, this study aimed to evaluate the usefulness of MC-SLO in detecting MA in DR compared with that of OCTA and CFP. The study found that MC-SLO had superior detection rate of MA compared with the two other methods but could also detect clinically significant MA.

## Results

### Subjects

Demographics of the participants are shown in Table 1. The study enrolled 54 eyes of 35 cases (men, 37 eyes of 25 cases; women, 17 eyes of 10 cases). The mean age was 64.5 ± 1.24 years. The mean best corrected visual acuity (logMAR) was 0.239 ± 0.043. The mean refractive error and axial length were − 1.24 ± 0.31 diopter and 24.0 ± 0.145 mm, respectively. There were 33 phakic and 21 pseudophakic eyes. Eleven cases (31.4%) had a history of smoking. Mean HbA1c was 7.36 ± 0.18%. We excluded cases that underwent any treatment for DME within one month of this study; however, 23 eyes had a treatment history, including 12 eyes with panretinal photocoagulation and 9 eyes with anti-VEGF therapy.

**Table 1:** Demographics of participants.

	Mean ± SE	Range
Age	64.5 ± 1.24	44 to 82
Right/left	24/30	
Sex (male/female)	25/10	
BCVA (logMAR)	0.239 ± 0.043	− 0.079 to 1.15
Refractive error	− 1.24 ± 0.31	− 5.83 to 4
Axial length (mm)	24.0 ± 0.145	21.9 to 26.2
Phakic/pseudophakic	33/21	
Smoking history (yes/no)	11/24	
Duration of diabetes (years)	12.9 ± 1.36	3 to 41
HbA1c (%)	7.36 ± 0.18	5.7 to 10.9
Treatment history (yes/no)^a^	23/31	
PRP	12	
Anti-VEGF therapy	9	
Vitrectomy	3	
STTA	3	

*SE* standard error, *BCVA* best corrected visual acuity, *PRP* panretinal photocoagulation, *VEGF* vascular endothelial growth factor, *STTA* subtenon triamcinolone acetonide injection.

^a^There is a history of multiple treatments in some cases.

### Sensitivity and positive predictive value for MAs in CFP, OCTA, and MC-SLO

We detected 1244 MAs by FA, which was the ground truth for MA detection in this study, in all cases. The number of objects that we judged as MAs included real MAs and MA-like objects that could not be confirmed as true MAs in FA images (i.e., objects that were mistakenly judged as MAs), was 117 in CFP, 304 in OCTA, and 642 in MC-SLO. The number of real MAs confirmed by FA images was 56 in CFP, 160 in OCTA, and 444 in MC-SLO.

The mean number of MAs we detected in FA images was 23.04 ± 3.53. The mean number of objects we judged as MAs was 2.17 ± 0.35 in CFP, 5.63 ± 0.56 in OCTA, and 11.9 ± 1.70 in MC-SLO. In addition, the mean number of real MAs confirmed by FA images was 1.04 ± 0.203 in CFP, 2.96 ± 0.35 in OCTA, and 8.22 ± 1.29 in MC-SLO.

**Figure 1:**
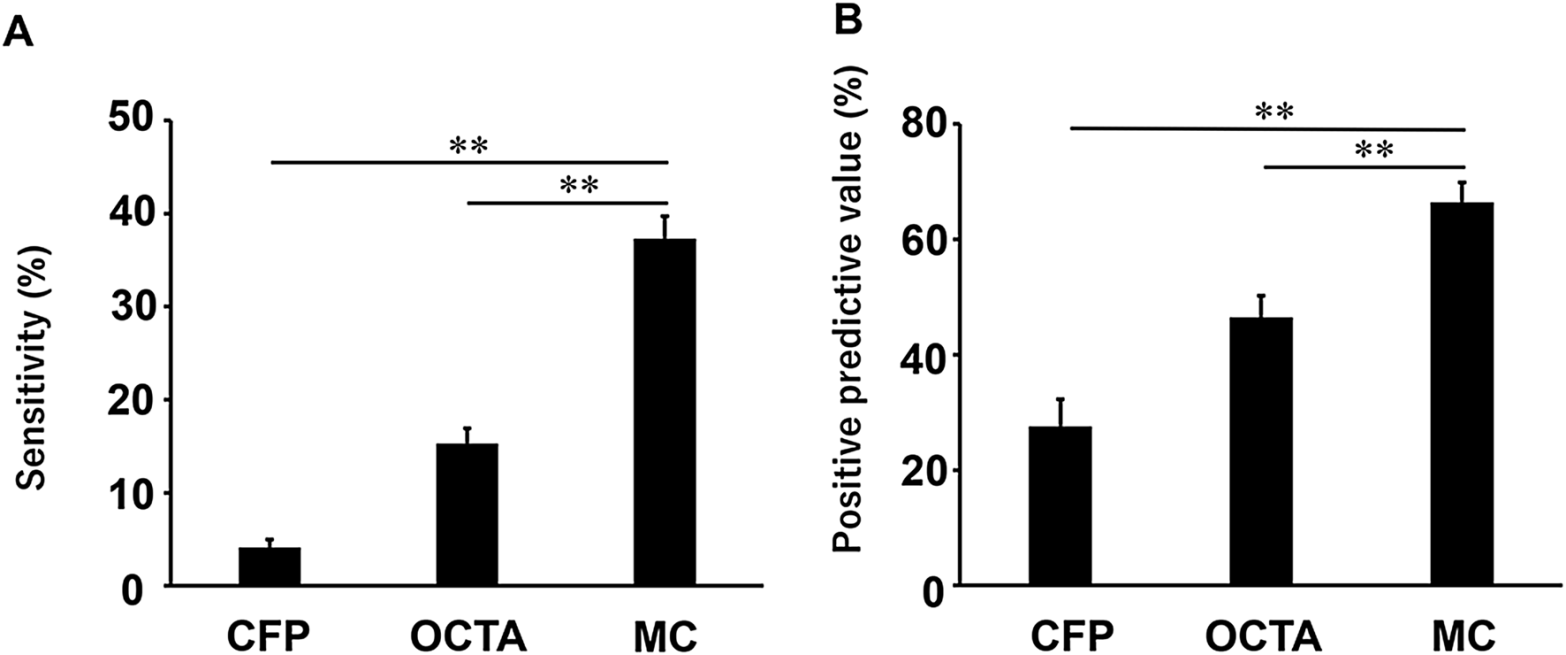
Sensitivity and positive predictive value of MAs in CFP, OCTA, and multicolor (MC) scanning laser ophthalmoscopy. The sensitivity of MAs was 4.12 ± 0.82% in CFP, 15.3 ± 1.63% in OCTA, and 37.3 ± 2.41% in MC-SLO (**A**). The positive predictive value of MAs was 27.6 ± 4.68%, 46.4 ± 3.81%, and 66.4 ± 3.40% in CFP, OCTA, and MC-SLO, respectively. (B) The sensitivity and positive predictive value of MC-SLO were significantly higher than those of OCTA and CFP (P < 0.01, Steel–Dwass test).

Therefore, the sensitivity was found to be 4.12 ± 0.82% in CFP, 15.3 ± 1.63% in OCTA, and 37.3 ± 2.41% in MC-SLO (Fig. 1A). The positive predictive value was 27.6 ± 4.68%, 46.4 ± 3.81%, and 66.4 ± 3.40% in CFP, OCTA, and MC-SLO, respectively (Fig. 1B). As a result, the sensitivity and positive predictive value of MC-SLO were significantly higher than those of OCTA and CFP (P < 0.01, Steel–Dwass test, Fig. 1).

### Comparison of deep layer and full-thickness image in OCTA

The sensitivity of MA detection in deep layer images was 5.80 ± 0.96%, and that in full-thickness images was 16.6 ± 2.26% (Supplementary Figure S1A). The positive predictive value of MA detection in deep layer images was 31.5 ± 5.65%, and that in full-thickness images was 47.7 ± 4.96% (Supplementary Figure S1B). Both sensitivity and positive predictive value were significantly higher in the full-thickness images than in deep layer images (P < 0.01, Wilcoxon test).

### Comparison of the retinal thickness at the MAs with early and late dye leakage

**Figure 2:**
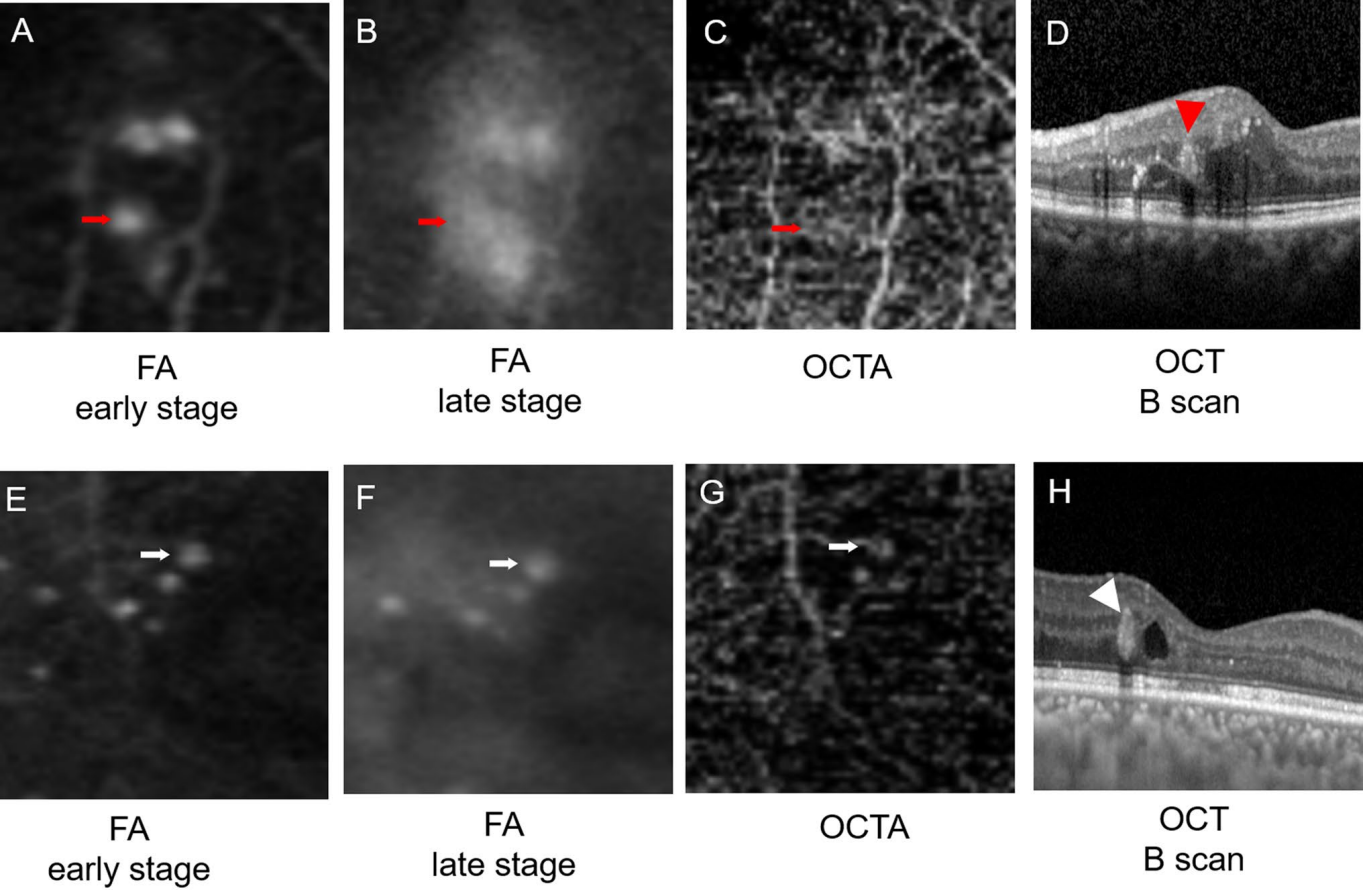
Representative cases of the eyes with MAs with early and late dye leakage. Images of the early phase (**A**,**E**) and late phase (**B**,**F**) of FA images, OCTA images (**C**, **G**),and OCT B-scan images (**D**,**H**) in cases of MAs with early leakage (**A**–**D**) and late leakage (**E**–**H**) are shown. The MA with early leakage (red arrow) was invisible on the OCTA image (**C**), but retinal edema was confirmed around the MA (white arrowhead) on the OCT B-scan (**D**). The MA with late leakage (white arrow) was detectable on the OCTA image (**G**), and retinal edema was not found around the MA (red arrowhead).

The retinal thickness at the MAs with early dye leakage (n = 65) was 433.8 ± 9.86 μm, and the retinal thickness at the MAs with late dye leakage (n = 85) was 373.0 ± 8.22 μm. The retinal thickness at the MAs with early leakage was significantly larger than that of the MAs with late leakage (P < 0.01, Mann–Whitney U test, Supplementary Figure S2A). Of these MAs, 67.8% and 16.1% were located in the inner nuclear layer (INL) and outer plexiform layer (OPL)/outer nuclear layer (ONL), respectively (Supplementary Table S2). A representative case is shown in Fig. 2.

### Comparison of sensitivities for the MAs with early, late, and no dye leakage in OCTA and MC-SLO

**Figure 3:**
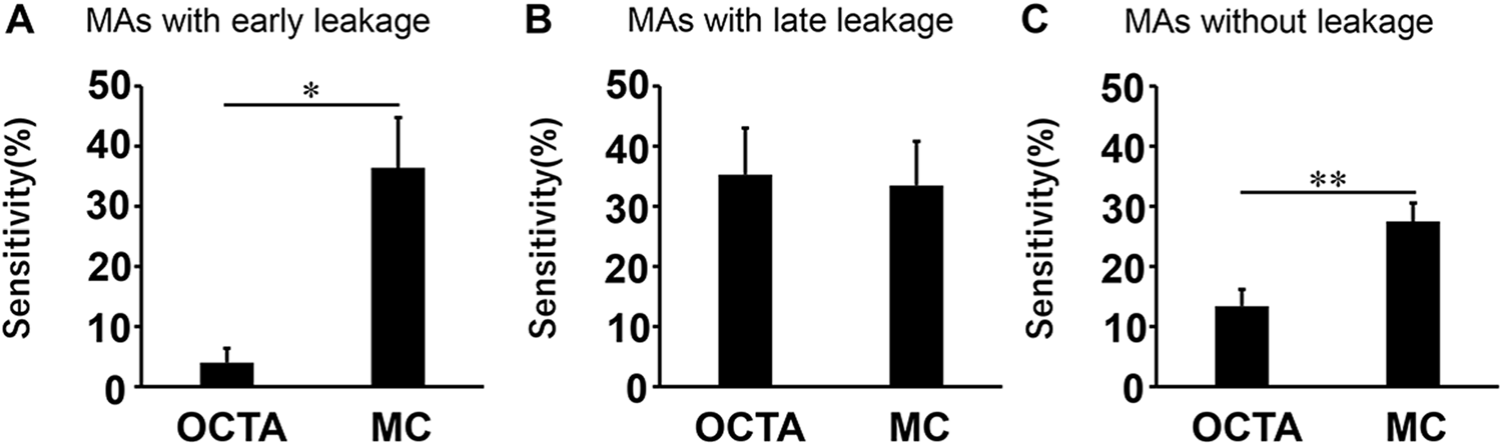
Sensitivities for MAs with early leakage, late leakage, and without leakage on OCTA and MC-SLO. Sensitivities for MAs with early (**A**), late (**B**), and no (**C**) leakage. Among all 23 eyes, sensitivity for MAs with early leakage (n = 65) was significantly greater in MC-SLO compared with that in OCTA (**A**). There was no significant difference between them for the sensitivity of MAs with late leakage (**B**). The sensitivity for MAs without leakage was significantly greater in MC-SLO compared with that in OCTA (**C**). *P < 0.05 and **P < 0.01.

The sensitivity for MAs with early leakage (n = 65) was 36.4 ± 8.39% in MC-SLO, which was significantly greater than 4.02 ± 2.37% in OCTA (P < 0.01, Wilcoxon test, Fig. 3A). The sensitivities for MAs with late leakage (n = 85) were 33.5 ± 7.34% in MC-SLO and 35.3 ± 7.71% in OCTA. There was no significant difference between them (P = 0.965, Wilcoxon test, Fig. 3B). The sensitivity for MAs without leakage was 27.4 ± 3.09% in MC-SLO, which was significantly greater than 14.6 ± 2.79% in OCTA (P < 0.01, Wilcoxon test, Fig. 3C).

### Comparison of the visibility of MAs in early and late dye leakage with OCTA and MC-SLO

Of the 62 MAs with early dye leakage, 47 (75.8%) were visible, whereas 15 (24.2%) were invisible in MC-SLO. In OCTA, 19 MAs (30.6%) were visible, whereas 43 (69.4%) were invisible. There was a significant difference in visibility of MAs between MC-SLO and OCTA (P < 0.01, Pearson's chi-square test, Supplementary Table S1). Of 90 MAs with late dye leakage, 76 MAs (84.4%) were visible, whereas 14 (15.6%) were invisible in MC-SLO. In OCTA, 75 MAs (83.3%) were visible, whereas 43 were invisible (16.7%). There was no significant difference between MC-SLO and OCTA (P > 0.05, Pearson’s chi-square test, Supplementary Table S1).

### Comparison of green/red ratio between MA and retinal hemorrhage in MC-SLO image

**Figure 4:**
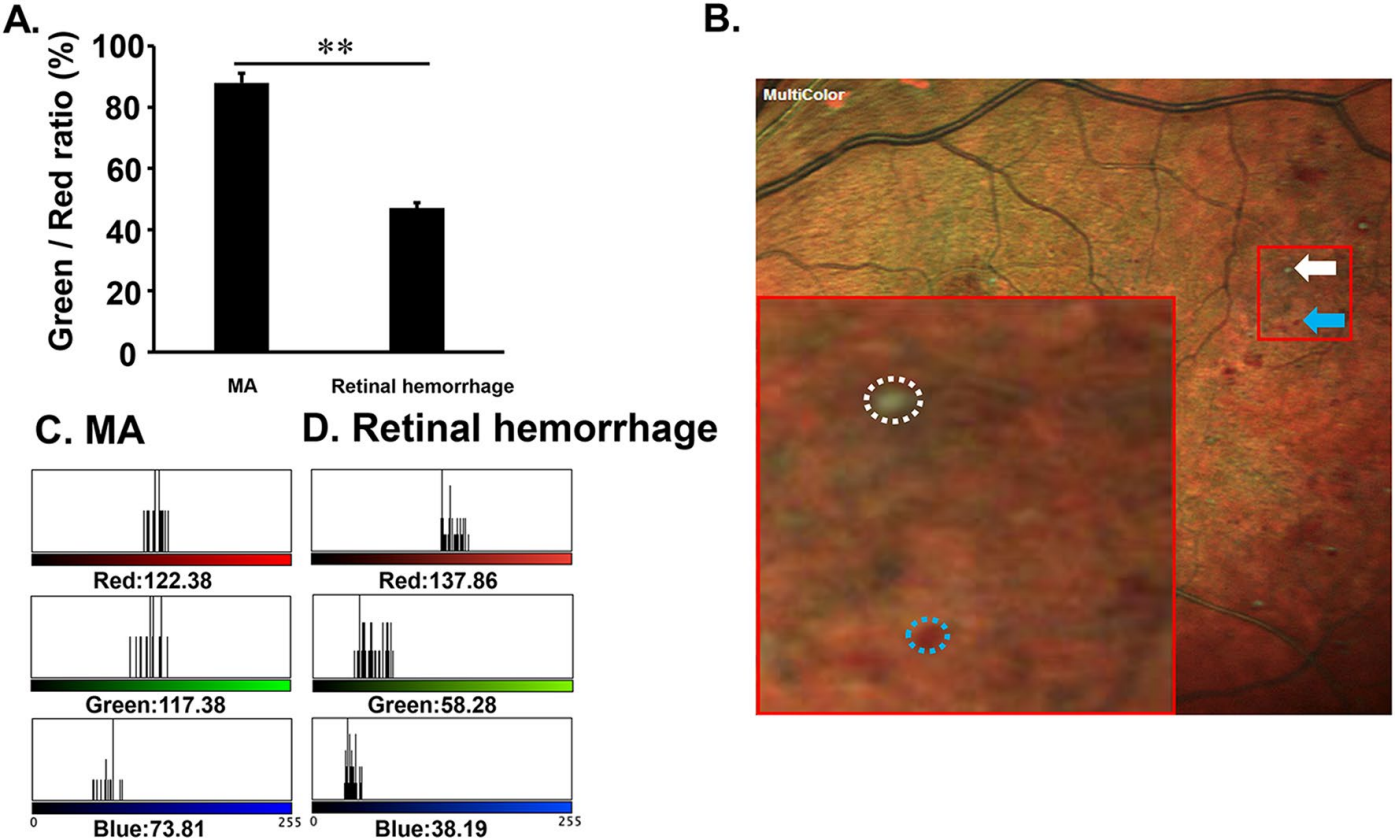
Color tone analysis of MAs and retinal hemorrhage. The green/red ratio of MAs was significantly higher than that of retinal hemorrhage. (**A**, **P < 0.01). (**B**) Representative image of MA (white dot circle and arrow) and retinal hemorrhage (blue dot circle and arrow) in MC-SLO. Results of color tone analysis in MA (**C**) and retinal hemorrhage (**D**). Note that the green tone in MA was higher than that in retinal hemorrhage.

We analyzed 32 MAs and 32 retinal hemorrhage dots. The green/red ratio of MAs was 87.96 ± 3.10%, and that of retinal hemorrhage was 47.05 ± 1.67% on the MC image. There was a significant difference in the ratio between MAs and retinal hemorrhage (P < 0.01, Mann–Whitney U test, Fig. 4).

## Discussion

The present results showed that MC-SLO had significantly greater detection sensitivity and positive predictive value of MAs than OCTA in the 6 × 6-mm image area.

The MC-SLO image has a digital depth resolution of 3.5 μm/pixel and one scan interval of 14 μm^27^. In contrast, the OCTA 6 × 6-mm scan has a horizontal direction of 11.719 μm and vertical direction of 23.438 μm, which was comparable with MC-SLO. Thus, the difference in the scan interval between machines did not seem to be a decisive factor for the current results.

In terms of appearance, MC-SLO showed the characteristic findings of MA. As in earlier studies^28^, most MAs had a characteristic finding, such as the central green dot with peripheral red color in MC-SLO. This feature made it easy to detect MA and distinguish it from retinal hemorrhages. Hemorrhagic dots showed more reddish color and less green color. According to pathological studies, MAs have a diameter of 50–100 μm^29–31^, and proliferation and degeneration of vascular endothelial cells are present in MAs^32–34^.

Fibrotic connective tissue, including thickened blood vessel wall, appears green in MC-SLO. Moreover, the red blood cell components in MA appear red^28^. Thus, it is feasible that the central green color of MAs reflects fibrotic changes and thickened vessel walls in MC-SLO. The second one was related to the mechanism for creating images in MC-SLO. In creating images, the MC-SLO gathers the horizontally reflected lights of the irradiated laser with a detector and calculates the signal strength to create an image.

**Figure 5:**
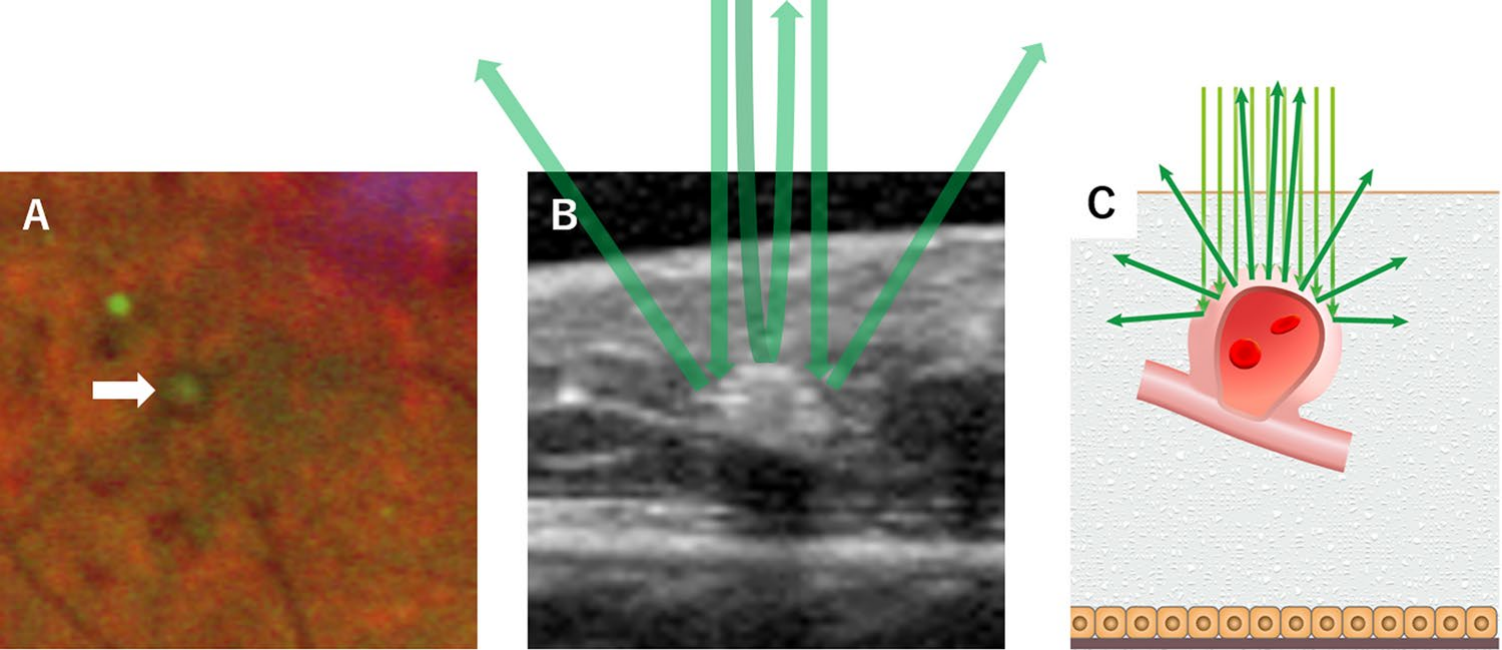
Schematic image of the characteristic finding of MAs in MC-SLO. Representative MA image in MC-SLO (**A**). MA was shown with a central green dot and peripheral red color. The green laser is strongly depicted in the central part of MA where the green laser is strongly reflected (**B**,**C**).

When the green wavelength light irradiates to the retina, a strong signal is reflected from the apex of MA toward the detector, but the vertical component of the reflected light from the side of MA became attenuated. As a result, the center of MAs appears green, and the surrounding area appears red, reflecting red blood cells in MC-SLO. Indeed, it is difficult to distinguish MA from retinal hemorrhage in regular CFP. However, it would be easily distinguished between two lesions; retinal hemorrhage reveals only a red color tone, and MAs have the aforementioned color tone pattern in MC-SLO (Fig. 5).

In contrast, OCTA had a low MA detection rate with 12.3%, which seemed lower than that in earlier studies, from 41 to 62% in 3 × 3-mm OCTA image^17–20^. It is possibly because a 6 × 6-mm image was used in this study, which is more commonly used than 3 × 3-mm image in clinical practice. The interval space of 6 × 6-mm image in OCTA is 11.719 μm in lateral scan, and that of 3 × 3 mm image is 5.859 μm. Similarly, the horizontal scan interval was 23.438 μm in 6 × 6-mm image and that of 3 × 3-mm image^20^ was 11.719 μm. The larger scan mesh made the detection rate lower. MC-SLO is not affected by that factor. Another reason was the difference in the analysis between this study and a previous study. In previous studies, the detection rate of MA was examined by superimposing (merging) FA and OCTA images^17, 19,20,35^. However, our raters evaluated the MAs in OCTA image separately, and the other rater calculated the detection rate after the primary analysis. Indeed, Carlo et al. studied the detection rate of MAs in OCTA with a method similar to ours^36^. They showed that the detection rate of microvascular abnormalities was 26.1%, which was comparable with our result.

Pathological research has shown that MA is a dilation of capillaries, predominantly in the central retina and mostly originating from the deep capillary plexus^33,34^. Similarly, using OCT B-scans, 67.8% and 80.3% of MAs were observed to be localized in the INL in this study and in Horii et al.’s study^37^, respectively (Supplementary Table S2). However, Parrulli et al. reported that Topcon OCTA, which is similar to the method used in the present study, detected many MAs (57%) in the deep (IPL-OPL) images but also detected 43% of MAs in the superficial (ILM-IPL) images, although there were differences in the results between the devices^38^. They also showed that more MAs were detectable when the two layers were combined. As shown in Supplementary Figure S2, we were able to detect more MAs in the full-thickness OCTA images than in the deep layer OCTA images. Thus, full-thickness OCTA images were used in the present study. The difference between the layers analyzed in the studies could affect the variation in the results.

Theoretically, an OCTA image is monochromatic and constructed by the movement of red blood cells. Thus, it could show not only MAs but also the surrounding capillaries. This feature might make it difficult to distinguish MAs from surrounding capillaries (representative images are shown in Supplementary Figure S3).

Even when normal capillaries bend or run vertically, it may be indistinguishable from MA. However, MAs could be visualized without showing normal capillaries in MC-SLO. Thus, the sensitivity and positive predictive value may be better in MC-SLO than in OCTA. Another advantage of MC-SLO is its superiority to detect clinically important MAs. Photocoagulation of MAs is performed when the MAs are the major cause of retinal edema^14^. Therefore, we defined MAs with early and late leakage by the findings in FA images and examined the sensitivity in MC-SLO and OCTA. First, the retinal thickness at the MA with early leakage was significantly greater than that of MAs with late leakage. Thus, MAs with early dye leakage are more important for the treatment of retinal edema than those with late dye leakage.

Interestingly, MAs with early dye leakage were found to be less detectable than MAs with late dye leakage in OCTA images. Further examination revealed that MA with early dye leakage was more signal-less than MA with late dye leakage in OCTA. There are two possible reasons as follows: first, in an MA with early dye leakage, the movement of red blood cells would be slow inside the MA, and thus, it may not be displayed as a blood flow signal at the current OCTA scan rate.

Nakao et al. studied the appearance of MA in AO-SLO and OCTA. They reported that MA with turbulence of blood cells was detectable in OCTA^35^. Thus, leaking pattern is thought to be diffuse and multidirectional in the MA with active leakage. These features might slow down blood flow, resulting in fewer signals of the MAs in OCTA. Second, a majority of the MAs with early leakage are associated with retinal edema. Retinal edema may prevent detection of MAs^37,39^. In contrast, the detection rate of MAs with early leakage was not decreased in MC-SLO. The reason is that MC-SLO captures only the “morphology” of MAs and is not affected by blood flow as OCTA.

There are several limitations of this study. First, this is a retrospective study with a relatively small number of cases, and the surveyed area is limited to 6 × 6 mm. Second, we evaluated images by objective methods as described. Although analyses were performed by experienced examiners, this might affect the results. It is necessary to be cautious of these limitations when interpreting and generalizing the present findings.

Third, this study included phakic and pseudophakic eyes. Intermediate opacities, including cataracts, are known to affect image quality to some extent^40–42^. Therefore, we excluded eyes with poor image quality owing to intermediate opacity, but the condition of the lens may have possibly affected the results. Finally, Arrigo et al. reported that 20% of MAs appear red dots in MC-SLO and these MAs correspond to type 1 MA, which are MAs with normal vascular endothelium and no pericytes, but with extensive accumulation of monocyte and polymorphonuclear cells in the lumen as per Stitt et al.’s histological MA classification^28,34^. We started this study with the understanding that MAs would be depicted with a green center in MC-SLO. Since the method of analysis and the background of the patients in our study differed from those in the paper by Arrigo et al. (mean age: 55 years in this study vs. 64.5 years in Arrigo et al.’s study, mean logMAR visual acuity: 0.6 in this study vs. 0.2 in Arrigo et al.’s study), the percentage of MAs that appear red dotsin our patients is unclear. However, it is possible that type 1 MAs that appear red were not included.

In conclusion, MC-SLO has higher sensitivity and positive predictive value for MA detection than OCTA. Especially for MAs with active leakage, which is clinically important for DME treatment, MC-SLO could detect MAs better than OCTA. Thus, it is suggested to use MC-SLO for managing eyes with diabetic retinopathy. FA remains to be the gold standard for MA detection; however, due to its invasiveness, MC-SLO is useful in cases where performing FA is difficult or when repeated evaluations are needed, such as for the evaluation of the treatment effect for DME.

## Methods

### Ethics statement

All procedures used in this study confirmed and complied with the tenets of the Declaration of Helsinki and were approved by the Ethics Committee of Kagoshima University Hospital. The committee considered that a written informed consent was not necessary due to the retrospective nature of the study.

### Study design, subjects, and examination method

This was a retrospective cross-sectional study. Consecutive patients with DR who visited Kagoshima University Hospital between January 2016 and May 2019 and underwent FA, MC-SLO (imaging range, 30°), OCTA (6 × 6-mm map, Triton, Topcon, Japan), and CFP (Triton) were included. Ocular examination was performed within 1 week. Eyes that received DME treatment within one month of the aforementioned examinations and eyes without clear images due to media opacities (such as cataract, which is known to affect the quality of fundus images^40–42^) were excluded from analysis. FA and MC-SLO images were taken using a Spectralis HRA (Heidelberg Engineering, Heidelberg, Germany) with at least 30 averaged images for MC-SLO. OCTA images were obtained with a central wavelength of 1050 nm, an acquisition speed of 100,000 A-scans/s, an axial resolution of 7 μm, and a transverse resolution of 20 μm. Scans were cubes of a 6 × 6 mm dimension with each cube consisting of 320 clusters of 4 repeated B-scans centered on the fovea. The captured images were extracted as TIFF images of the highest quality. All subsequent analyses were performed on the same computer.

### Sensitivity and positive predictive value of MA detection in CFP, OCTA, and MC

**Figure 6:**
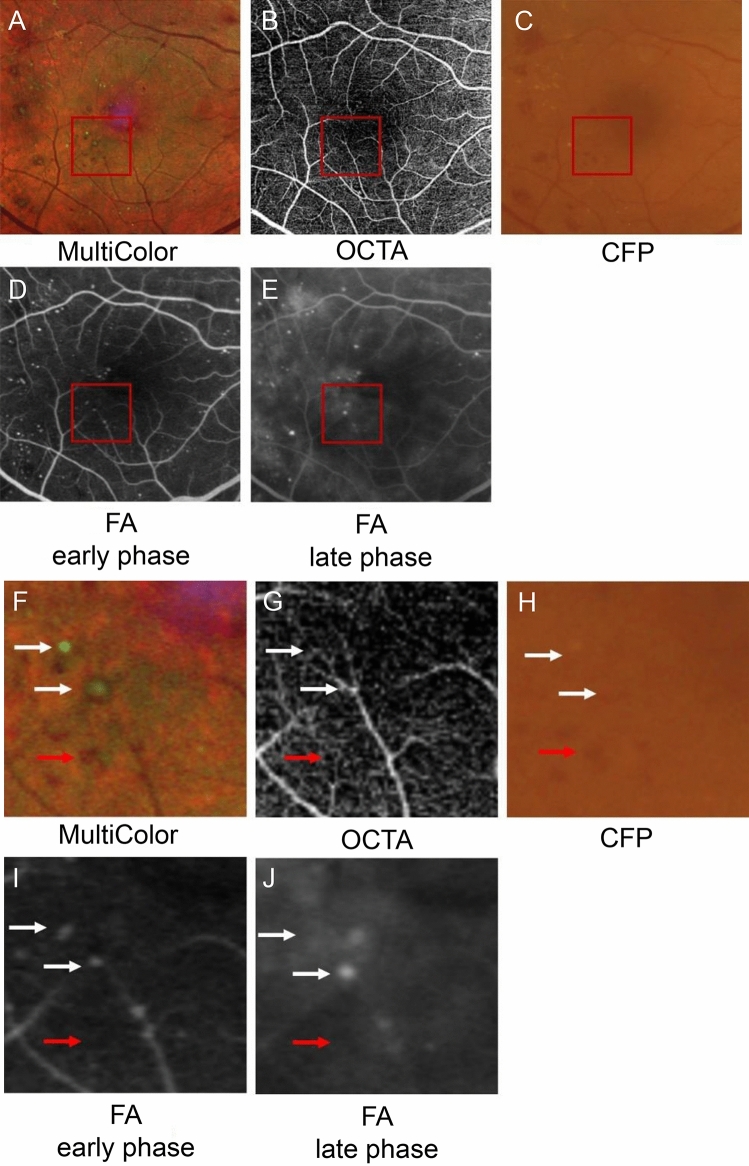
Comparison of MA and retinal hemorrhage in multicolor scanning laser ophthalmoscopy (MC-SLO), optical coherence tomography angiography (OCTA), color fundus photography (CFP), and fluorescein angiography (FA). Upper panels are 6 × 6-mm images of MC-SLO (**A**), OCTA (**B**), CFP (**C**), and early and late phases of FA (**D**,**E**). (**F**–**J**) Magnified images of the red squares in the upper images. Leakage of angiogenic dye was strong at the MA with upper white arrow in (**J**). Thus, the outline of MA could not be traced. Red arrow shows retinal hemorrhage.

The images of CFP and MC were cut in the same size as the image of 6 × 6 mm OCTA using Photoshop (Adobe Systems Inc., San Jose, USA). The ground truth of the presence of MA was determined using FA. On the basis of the study by Schreur et al.^19^ MAs in OCTA images were defined as hyporeflective, moderate, or hyperreflective spots with various morphologic patterns, including fusiform, saccular, curved, and rarely coiled shapes. MAs in MC-SLO images were also defined as green dot and green dot with peripheral red color partly based on the study by Arrigo et al.^28^. The presence of MAs in each image was determined by two examiners (T.S. and H.T.). Another independent evaluator (H.S.) calculated the sensitivity and positive predictive value based on the following formulas. True MA was determined using FA and used as the ground truth (Fig. 6).

Sensitivity of each method = number of true MAs in all MAs selected in each image/number of MAs in FA.

Positive predictive value of each method = number of true MAs in all MAs selected in each image/total number of MAs selected in the images.

### Comparison of MA evaluation between images of the deep layers of the retina and the whole retina by OCTA

Most MAs are known to be present in the deep layer of the retina, and many studies evaluated MAs using images of the deep layers of the retina obtained by OCTA^18,20^. Therefore, we compared the sensitivity of MAs between the images of the deep layer of the retina and the whole retina. Each 6 × 6 mm OCTA image was obtained using the embedded software in the OCTA machine. Two examiners (T.S. and H.T.) evaluated the MAs as described earlier. Another evaluator (H.S.) evaluated the true positive rate and its positive predictive value based on FA images. Furthermore, we analyzed the vertical location of these MAs using OCT B-scans. The number of MAs in each retinal layer, such as the nerve fiber layer, ganglion cell layer/inner plexiform layer, INL, and OPL/ONL, was counted. MAs for which determining the vertical location was difficult due to diffuse macular edema were excluded from the analysis. This analysis was performed according to the method previously reported by Horii et al.^37^.

### Differences in retinal thickness between MAs with early and late leakage

In FA, MAs with early dye leakage are supposed to leak more dye than those with late leakage. It is possible that the former is involved in the pathology of DME more significantly than the latter. To assess this hypothesis, we compared the retinal thickness around MAs with early, late, and no dye leakage.

**Figure 7:**
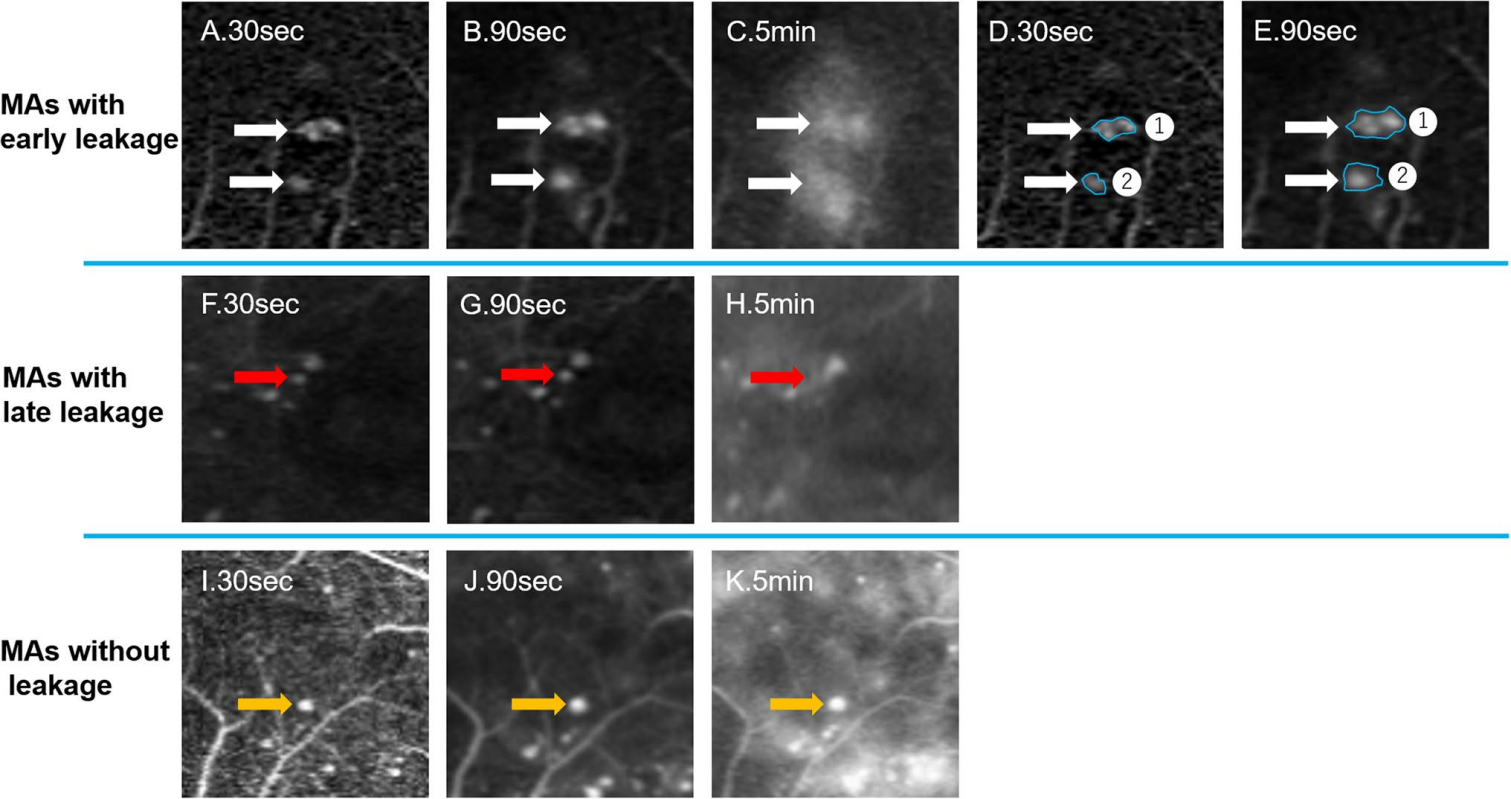
Representative images of three types of MAs with dye leakage in fluorescein angiography. (**A**–**E**) MAs with early dye leakage at different time points (white arrows). Areas of MA with upper and lower arrows at 30 s were 29,375 and 6875 μm^2^, respectively, and enlarged to 109,538 and 38,769 μm^2^, respectively, at 90 s. (**F**–**H**) MAs with late dye leakage (red arrow). (**I**–**K**) MAs without dye leakage (yellow arrow).

To distinguish MAs with early dye leakage, we performed an evaluation based on the following criteria (Fig. 7):

1. The size of FA leakage at 30 s was defined as the basic leakage size.
2. The size of FA leakage at 105 ± 15 s was defined as the early leakage size.
3. The size of FA leakage at 270 ± 30 s was defined as the late leakage size.

MA with “early dye leakage” had an early leakage size more than three times larger than the basic leakage size.

MA with “late dye leakage” had a late leakage size more than three times larger than basic leakage size but did not satisfy the criteria of “early dye leakage”.

MA with “no leakage” had neither early nor late dye leakage.

The retinal thickness of the region of each MA was measured with embedded calipers in the OCT B-scan image, and the results were compared between groups. We also compared the sensitivities of the three aforementioned types of MAs in CFP, OCTA, and MC images using the aforementioned methods.

### Comparison of MAs in the visibility of dye leakage in OCTA and MC-SLO images

To investigate whether the degree of fluorescence leakage from MAs affects the detection of MAs in OCTA and MC-SLO, the detection rates of MAs with early and late dye leakage in each image were compared. Two examiners (T.S. and H.T.) detected MAs in OCTA (6 × 6 mm) and MC-SLO images in cases of MAs with early and late dye leakage. The sensitivity (detection rate) of both types of MAs was calculated, and the results were compared between OCTA and MC-SLO.

### Comparison of color tone between MA and retinal hemorrhage on MC-SLO images

Because many MAs appear as green dots and retinal hemorrhages appear as red dots on MC-SLO images, the ratio of green and red tones between MAs and retinal hemorrhage was analyzed. From eight eyes containing four or more MAs and retinal hemorrhage, four of each finding were selected on the basis of size order, resulting in a total of 32 spots, and then extracted and analyzed. The color tone of the findings was analyzed using the Wayne Resband mode in ImageJ software (NIH, Bethesda, MD, USA) to obtain the values of red, green, and blue tones. The green/red ratios were calculated and compared.

### Statistical analysis

Statistical analyses were performed using SPSS for Windows software (SPSS, Inc., IBM, Somers, NY, USA) or EZR software (Saitama Medical Center, Jichi Medical University, Saitama Japan)^43^.

The Steel–Dwass test was used to compare the sensitivity (true positive rate) of CFP, OCTA, and MC-SLO with the positive predictive value. The Wilcoxon test was used to compare the sensitivity (true positive rate) for detection of MAs in the deep layer mode and full retina mode of OCTA (true positive rate) and the true positive rate for the detection of MAs with active, mild, and no leakage. The Mann–Whitney U test was used to compare the retinal thickness of leaking MAs with early, late, and no leakage. The green/red ratios on MC-SLO images of MAs and retinal hemorrhage were analyzed. A P value < 0.05 was considered to be statistically significant.

## Supplementary Information

Supplementary Information 1.

Supplementary Information 2.

Supplementary Information 3.

Supplementary Information 4.

Supplementary Information 5.
