# Supplementary material for: Comparison of multicolor scanning laser ophthalmoscopy and optical coherence tomography angiography for detection of microaneurysms in diabetic retinopathy
Source: Sci Rep. 2021 Aug 23;11:17017. doi: 10.1038/s41598-021-96371-y (PMC8382757; doi:10.1038/s41598-021-96371-y)
Supplement: Supplementary file 1 — Supplementary Information 1. [file 41598_2021_96371_MOESM1_ESM.docx]

**Comparison of multicolor scanning laser ophthalmoscopy and optical coherence tomography angiography for detection of microaneurysms in diabetic retinopathy**

Takato Sakono, Hiroto Terasaki, Shozo Sonoda, Ryoh Funatsu, Hideki Shiihara, Eisuke Uchino, Toshifumi Yamashita, Taiji Sakamoto

Department of Ophthalmology, Kagoshima University Graduate School of Medical and Dental Sciences, Kagoshima, Japan

**Supplementary Figure S1**

**
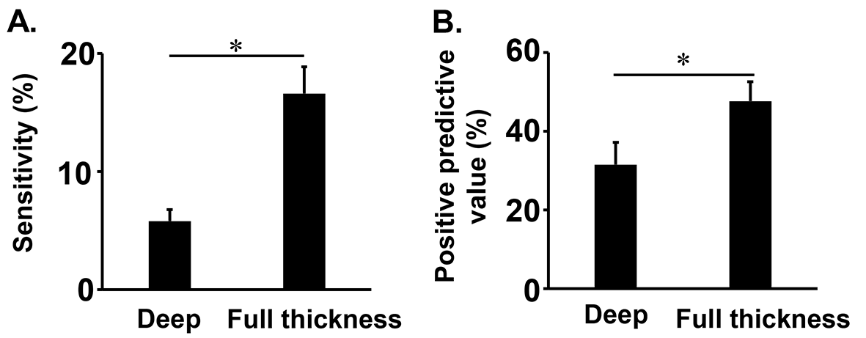
**

**Sensitivity and positive predictive value of MA in OCTA in deep layer and full-thickness images.**

The sensitivity of MA detection in deep layer images was 5.80 ± 0.96%, and that in full-thickness images was 16.6 ± 2.26% (A). The positive predictive value of MA detection in deep layer images was 31.5 ± 5.65%, and that in full-thickness images was 47.7 ± 4.96% (B). Both sensitivity and positive predictive value were significantly higher in the full-thickness images than in deep layer images (P < 0.01, Wilcoxon test).
