# Supplementary material for: Comparison of multicolor scanning laser ophthalmoscopy and optical coherence tomography angiography for detection of microaneurysms in diabetic retinopathy
Source: Sci Rep. 2021 Aug 23;11:17017. doi: 10.1038/s41598-021-96371-y (PMC8382757; doi:10.1038/s41598-021-96371-y)
Supplement: Supplementary file 2 — Supplementary Information 2. [file 41598_2021_96371_MOESM2_ESM.docx]

**Comparison of multicolor scanning laser ophthalmoscopy and optical coherence tomography angiography for detection of microaneurysms in diabetic retinopathy**

Takato Sakono, Hiroto Terasaki, Shozo Sonoda, Ryoh Funatsu, Hideki Shiihara, Eisuke Uchino, Toshifumi Yamashita, Taiji Sakamoto

Department of Ophthalmology, Kagoshima University Graduate School of Medical and Dental Sciences, Kagoshima, Japan

**Supplementary Figure S2**

**
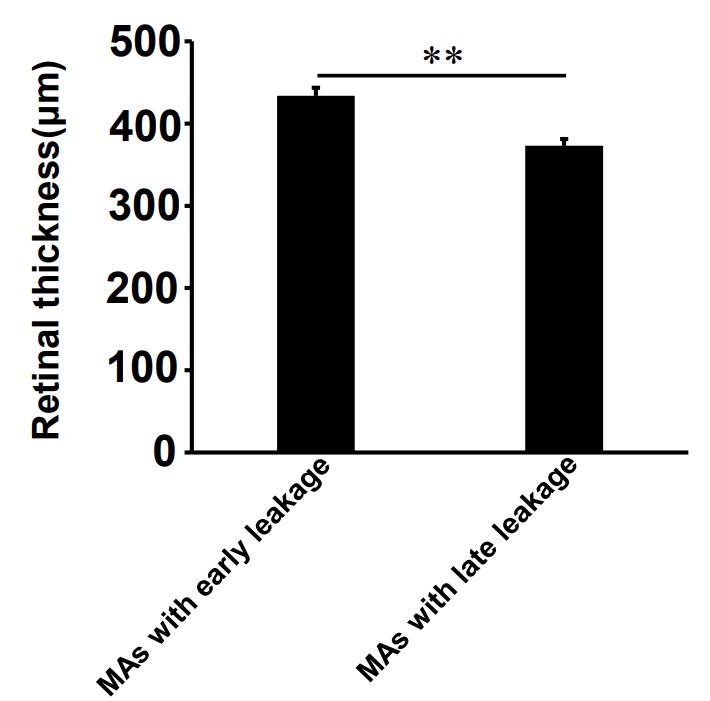
**

**Retinal thickness at the MAs with early and late leakage.**

The retinal thickness at the MAs with early dye leakage (n = 65) was 433.8 ± 9.86 μm, and the retinal thickness at the MAs with late dye leakage (n = 85) was 373.0 ± 8.22 μm. The retinal thickness at the MAs with early leakage was significantly larger than that of the MAs with late leakage (**P < 0.01, Mann–Whitney U test).
