# Supplementary material for: Comparison of multicolor scanning laser ophthalmoscopy and optical coherence tomography angiography for detection of microaneurysms in diabetic retinopathy
Source: Sci Rep. 2021 Aug 23;11:17017. doi: 10.1038/s41598-021-96371-y (PMC8382757; doi:10.1038/s41598-021-96371-y)
Supplement: Supplementary file 3 — Supplementary Information 3. [file 41598_2021_96371_MOESM3_ESM.docx]

**Comparison of multicolor scanning laser ophthalmoscopy and optical coherence tomography angiography for detection of microaneurysms in diabetic retinopathy**

Takato Sakono, Hiroto Terasaki, Shozo Sonoda, Ryoh Funatsu, Hideki Shiihara, Eisuke Uchino, Toshifumi Yamashita, Taiji Sakamoto

Department of Ophthalmology, Kagoshima University Graduate School of Medical and Dental Sciences, Kagoshima, Japan

**Supplementary Figure S3**

**
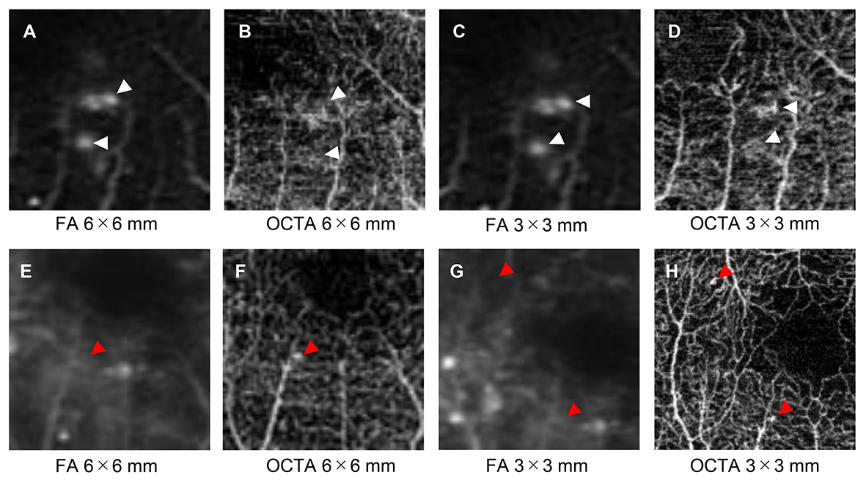
**

**False positive and false negative detection of MA in OCTA.**(A, E) FA 6 × 6 mm, (B, F) OCTA 6 × 6 mm, (C, G) FA 3 × 3 mm, (D, H) OCTA 3 × 3 mm
In the upper row, MA (white arrow head) can be recognized by FA in A and C, but it is difficult to detect in both 6 × 6 mm (B) and 3 × 3 mm (D) OCTA images.
In the lower row, an MA-like image (red arrowhead) is shown in OCTA 6 × 6 mm (F) and 3 × 3 mm (H), but MAs could not be confirmed by FA.
