# Supplementary material for: Comparison of multicolor scanning laser ophthalmoscopy and optical coherence tomography angiography for detection of microaneurysms in diabetic retinopathy
Source: Sci Rep. 2021 Aug 23;11:17017. doi: 10.1038/s41598-021-96371-y (PMC8382757; doi:10.1038/s41598-021-96371-y)
Supplement: Supplementary file 4 — Supplementary Information 4. [file 41598_2021_96371_MOESM4_ESM.docx]

**Comparison of multicolor scanning laser ophthalmoscopy and optical coherence tomography angiography for detection of microaneurysms in diabetic retinopathy**

Takato Sakono, Hiroto Terasaki, Shozo Sonoda, Ryoh Funatsu, Hideki Shiihara, Eisuke Uchino, Toshifumi Yamashita, Taiji Sakamoto

Department of Ophthalmology, Kagoshima University Graduate School of Medical and Dental Sciences, Kagoshima, Japan

**Supplementary Table S1. Comparison of appearance of the leaking MA from the early and latter stages in MC and OCTA**

MA with early leakage

|  | MC | OCTA |
| --- | --- | --- |
| Visible | 47/62 (75.8%) | 19/62 (31.4%) |
| Invisible | 15/62 (24.2%) | 43/62 (69.4%) |

MC vs OCTA, P < 0.01, Pearson's chi-square test

MA with late stage

|  | MC | OCTA |
| --- | --- | --- |
| Visible | 76/90 (84.4%) | 75/90 (83.3%) |
| Invisible | 14/90 (15.4%) | 15/90 (16.7%) |

MC vs OCTA, P > 0.05, Pearson's chi-square test

MA, microaneurysm; MC, multicolor; OCTA, optical coherence tomography angiography
