# Supplementary material for: Comparison of multicolor scanning laser ophthalmoscopy and optical coherence tomography angiography for detection of microaneurysms in diabetic retinopathy
Source: Sci Rep. 2021 Aug 23;11:17017. doi: 10.1038/s41598-021-96371-y (PMC8382757; doi:10.1038/s41598-021-96371-y)
Supplement: Supplementary file 5 — Supplementary Information 5. [file 41598_2021_96371_MOESM5_ESM.docx]

**Comparison of multicolor scanning laser ophthalmoscopy and optical coherence tomography angiography for detection of microaneurysms in diabetic retinopathy**

Takato Sakono, Hiroto Terasaki, Shozo Sonoda, Ryoh Funatsu, Hideki Shiihara, Eisuke Uchino, Toshifumi Yamashita, Taiji Sakamoto

Department of Ophthalmology, Kagoshima University Graduate School of Medical and Dental Sciences, Kagoshima, Japan

**Vertical location of microaneurysms in Retinal layer**

| **Retinal layer** | **Number of MAs** |
| --- | --- |
| NFL | 0 (0 %) |
| GCL/IPL | 14 (16.1 %) |
| INL | 59 (67.8 %) |
| OPL/ONL | 14 (16.1 %) |

MA; microaneurysms, NFL; nerve fiver layer, GCL; ganglion cell layer, IPL; inner plexiform layer, INL; inner nuclear layer, OPL; outer plexiform layer, ONL: outer nuclear layer
